# EEG spectral analysis of attention in ADHD: implications for neurofeedback training?

**DOI:** 10.3389/fnhum.2014.00611

**Published:** 2014-08-21

**Authors:** Hartmut Heinrich, Katrin Busch, Petra Studer, Karlheinz Erbe, Gunther H. Moll, Oliver Kratz

**Affiliations:** ^1^Department of Child and Adolescent Mental Health, University Hospital of ErlangenErlangen, Germany; ^2^Heckscher-KlinikumMünchen, Germany; ^3^Practice of Child and Adolescent PsychiatryBamberg, Germany

**Keywords:** neurofeedback, ADHD, EEG, spectral analysis, attention, brain-behavior-relationship, subtypes

## Abstract

**Objective:** In children with attention-deficit/hyperactivity disorder (ADHD), an increased theta/beta ratio in the resting EEG typically serves as a rationale to conduct theta/beta neurofeedback (NF) training. However, this finding is increasingly challenged. As NF may rather target an active than a passive state, we studied the EEG in a condition that requires attention.

**Methods:** In children with ADHD of the DSM-IV combined type (ADHD-C; *N* = 15) and of the predominantly inattentive type (ADHD-I; *N* = 9) and in typically developing children (*N* = 19), EEG spectral analysis was conducted for segments during the attention network test (ANT) without processing of stimuli and overt behavior. Frontal (F3, Fz, F4), central (C3, Cz, C4) and parietal (P3, Pz, P4) electrodes were included in the statistical analysis. To investigate if EEG spectral parameters are related to performance measures, correlation coefficients were calculated.

**Results:** Particularly in the ADHD-C group, higher theta and alpha activity was found with the most prominent effect in the upper-theta/lower-alpha (5.5–10.5 Hz) range. In the ADHD-I group, a significantly higher theta/beta ratio was observed at single electrodes (F3, Fz) and a tendency for a higher theta/beta ratio when considering all electrodes (large effect size). Higher 5.5–10.5 Hz activity was associated with higher reaction time variability with the effect most prominent in the ADHD-C group. A higher theta/beta ratio was associated with higher reaction times, particularly in the ADHD-I group.

**Conclusions:** (1) In an attention demanding period, children with ADHD are characterized by an underactivated state in the EEG with subtype-specific differences. (2) The functional relevance of related EEG parameters is indicated by associations with performance (reaction time) measures. (3) Findings provide a rationale for applying NF protocols targeting theta (and alpha) activity and the theta/beta ratio in subgroups of children with ADHD.

## Introduction

Theta/beta training belongs to the neurofeedback (NF) protocols which are frequently applied in children with ADHD; for review see Arns et al. (2014) and Gevensleben et al. (2014). In theta/beta training, the aim is to decrease theta activity and to increase activity in the beta band of the EEG or to decrease the theta/beta ratio with feedback being calculated typically from electrode Cz. In randomized controlled trials, it has been found to be superior in reducing the children’s inattentive, hyperactive and impulsive behavior (medium effect sizes) compared to computerized attention training (Gevensleben et al., 2009a) and EMG biofeedback (Bakhshayesh et al., 2011).

Specificity of training effects is further supported by findings at the neurophysiological level (Gevensleben et al., 2009b). Higher baseline theta activity in the resting EEG (recorded in an eyes open condition) over centro-parietal regions was associated with a larger reduction of the severity of ADHD symptoms after theta/beta training and larger decreases of theta activity from pre- to post-training were accompanied by larger clinical improvements. These findings also indicate that it should be possible to derive EEG-based indication criteria for which children theta/beta training may be more appropriate.

As a rationale for applying theta/beta training in ADHD, authors typically referred to findings from resting EEG studies comparing children with ADHD to typically developing controls (see e.g., Heinrich et al., 2007).

### Resting EEG studies in ADHD

A series of resting EEG studies in ADHD (eyes open and eyes closed condition) have been conducted since the 1980s and reviewed e.g., in Barry et al. (2003). Consistently, elevated levels of theta activity and reduced relative levels of beta and alpha activity (corresponding to increased theta/beta and theta/alpha ratios) were found compared to typically developing children. Slow activity was described to have a fronto-central distribution although group differences were most prominent over posterior regions (Banaschewski and Brandeis, 2007). Deviances appeared to be more prominent in the DSM-IV combined type of ADHD compared to the predominantly inattentive subtype.^1^

The theta/beta ratio measured at Cz was reported to discriminate reliably between children with ADHD and controls (classification rate: *ca*. 90%; Monastra et al., 1999; Snyder et al., 2008). On the other hand, Barry et al. (2003) stated EEG heterogeneity in ADHD and suggested to define EEG-based subtypes of ADHD.

Applying theta/beta training was thought to “normalize” the cortical slowing. However, recent studies question if the major part of children with ADHD are actually characterized by an increased theta/beta ratio in the resting EEG. Arns et al. (2013) conducted a meta-analysis studying theta/beta ratio in an eyes-open condition at electrode Cz. Including nine studies with about 1200 children and adolescents with ADHD and about 500 children without ADHD, they found a medium effect size of 0.62 (age range from 6 to 18 years). However, the authors argued that this number is misleading as *post hoc* analysis revealed a decreasing difference in theta/beta ratio across years due to an increasing theta/beta ratio for the non-ADHD (control) participants.

This point of view is further supported by two studies, which were published after this meta-analysis and did not find differences between children with ADHD and typically developing children in any frequency band considered (Liechti et al., 2013; Buyck and Wiersema, 2014). However, subdividing the ADHD group revealed increased theta/beta ratios in children and adults of the predominantly inattentive subtype in Buyck and Wiersema (2014) who analyzed EEG activity at midline electrodes.

### EEG studies in ADHD during task performance

Interpreting NF as a neurobehavioral approach, training rather targets an active than a passive state (Gevensleben et al., submitted). For example, training may also comprise trials combined with tasks (e.g., reading, listening). In this respect, it appears to be more relevant to consider the EEG during task processing though it has to be kept in mind that the resting EEG does not only reflect a trait but also a state marker (Hagemann et al., 2005).

Up to now, EEG profiles in ADHD during cognitive tasks have less often been studied. Monastra et al. (1999) did not only study the theta/beta ratio in a resting condition at single electrode Cz but also while children were reading, listening and drawing. For all conditions, the ADHD group was characterized by increased theta/beta ratios. In El-Sayed et al. (2002), increased slow activity (mainly over frontal electrodes) was found especially during an attention (continuous performance) task but also during eyes-open resting condition.

Loo and Smalley (2008) investigated familiality of spectral EEG measures in ADHD during resting and cognitive activation (sustained attention task) conditions. Effects were clearly stronger for the activation compared to the resting conditions and did not show topographic specificity. Sibling correlations of 0.6–0.7 were obtained for the theta, alpha and beta band. Theta and alpha power were associated with task performance (reaction time variabililty, omission errors). So, not only theta and beta activity but also alpha activity should be considered when studying EEG activity during an activation condition in the context of ADHD. However, in our opinion, two points were not realized in an optimal way in the study of Loo and Smalley (2008). First, associations between EEG and performance measures were not controlled for potential developmental effects and, second, we would prefer to analyze EEG segments reflecting an attentive state without processing of task-related information (stimuli) as event-related EEG components interfere with the spontaneous activity.

Lazzaro et al. (2001) reported increased pre-stimulus theta activity in children with ADHD during an oddball task. Theta activity correlated inter alia with the latency of the event-related potential component P3 indexing attention.

### Information about the dataset/objectives of the study

In the present case-control study, we conducted EEG spectral analysis in children with ADHD during an attentive state. For this analysis, we used a previously published dataset (Kratz et al., 2011). In Kratz et al. (2011), attentional processing was studied in children with ADHD during the attention network test (ANT). At the neural level (event-related potentials), deviant cue processing (reduced cue-P3) was the most prominent effect. The contingent negative variation (CNV)^2^ reflecting inter alia cognitive preparation processes was not found to be smaller—probably due to the younger age of this sample compared to other studies (e.g., Albrecht et al., 2013). Differences between ADHD subtypes (combined type vs. predominantly inattentive type) could be observed.

Using this dataset for the present analysis allowed to consider segments during the ANT reflecting a state of activation/(tonic) alertness and free of stimulus processing. We expected that children with ADHD show increased theta activity and/or an increase theta/beta ratio across the scalp surface during an attentive state serving as a rationale to apply related protocols in NF training.

We were also interested in comparing DSM-IV subtypes of ADHD. In order to learn more about the functional significance of the spectral EEG parameters, we studied associations (correlations) between these spectral EEG parameters and performance measures (particularly reaction time measures).

## Materials and methods

### Participants

Fifteen children with ADHD of the combined type (ADHD-C; according to DSM-IV criteria), nine children with ADHD of the predominantly inattentive subtype (ADHD-I) and 19 typically developing children were included in the study. Children had to be aged 8–11 years and to have a full-scale IQ of at least 80. All children had normal or corrected-to-normal vision. Adequate task performance in the ANT and sufficient EEG data quality was also necessary to be included in this study (for details see below). The three groups (ADHD-C, ADHD-I, controls) were comparable regarding age and sex (Demographic and clinical variables of the sample are summarized in Table 1). IQ was significantly lower in the ADHD-I group but IQ had no significant influence on the group-specific results as tested by comparing the ADHD-I group to a subgroup of typically developing children with comparable IQ; see also Kratz et al. (2011).

**Table 1:** **Sample characteristics and performance data of the attention network test**.

	**Children with ADHD**		**Controls (*N* = 19)**	**Statistics**
	**ADHD-C (*N* = 15)**	**ADHD-I (*N* = 9)**		
Age (months)	117.8 ± 12.0	112.6 ± 12.6	122.0 ± 11.9	*F*_(2,40)_ = 1.9, n.s.
IQ	114.3 ± 11.3	102.7 ± 11.6	114.7 ± 11.1	*F*_(2,40)_ = 4.4, *p* = 0.02
Sex (m/f)	10/5	8/1	15/4	χ^2^ = 1.65, n.s.
**German ADHD rating scale (FBB-HKS)**				
Total score	1.56 ± 0.37	1.34 ± 0.38	0.34 ± 0.22	*F*_(2,40)_ = 67.9, *p* < 0.001
Inattention	1.77 ± 0.44	1.81 ± 0.47	0.50 ± 0.33	*F*_(2,40)_ = 52.4, *p* < 0.001
Hyperactivity/impulsivity	1.42 ± 0.48	0.96 ± 0.38	0.21 ± 0.20	*F*_(2,40)_ = 46.9, *p* < 0.001
**Associated disorders**				
Oppositional defiant disorder	2	0	−	
Emotional disorder	1	1	−	
Dyslexia	1	2	−	
**Attention network test**				
Hits (correct responses)	171.5 ± 19.4	181.0 ± 5.8	175.1 ± 17.2	*F*_(2,40)_ = 0.9, n.s.
Reaction times—median (ms)	535.0 ± 99.7	643.3 ± 122.9	508.3 ± 70.3	*F*_(2,40)_ = 6.5, *p* = 0.004
Reaction time variability (ms)	142.6 ± 39.2	160.5 ± 48.5	112.0 ± 29.0	*F*_(2,40)_ = 6.0, *p* = 0.005

Patients were either recruited from a child and adolescent psychiatric practice in Bamberg (Germany) and took part in a medication trial (Kratz et al., 2012) or were recruited via the outpatient department of the Department of Child and Adolescent Mental Health at the University Hospital of Erlangen and participated in a NF trial (Gevensleben et al., 2009a). Baseline measurements (conducted before starting treatment) were considered for the present analysis. Diagnostics comprised a clinical interview conducted by a child and adolescent psychiatrist or a clinical psychologist. ADHD diagnoses were confirmed using the Diagnostic Checklist for Hyperkinetic Disorders/ADHD (Döpfner and Lehmkuhl, 2000). Patients had no comorbid diagnoses other than oppositional defiant disorder, emotional disorder and dyslexia. All children with ADHD included in this study were drug-naive. Typically developing children were recruited from the personal environment of employees of the clinic. For none of the children of the control group, parents reported a psychiatric or neurological disorder.

For all children, the German ADHD rating scale (FBB-HKS: Döpfner and Lehmkuhl, 2000) was assessed (filled out by parents). The FBB-HKS is a 20-item questionnaire related to the DSM-IV and ICD-10 criteria for ADHD (nine inattention items, seven hyperactivity items, four impulsivity items). Severity of each item is rated on a scale from 0 to 3. The questionnaire provides a total score (mean value of all 20 items) as well as subscores for inattention and hyperactivity/impulsivity. For the typically developing children included in the study, FBB-HKS scores (total score and subscales) were not more than one standard deviation above normative means. Control and ADHD groups differed on all FBB-HKS scales (*F*_(2,40)_ > 46.9; *p* < 0.001). For the two ADHD groups (ADHD-C vs. ADHD-I), the FBB-HKS total score (*t*_(22)_ = 1.38, n.s.) and the score for the inattention subscale (*t*_(22)_= −0.20, n.s.) were comparable. However, the score for the hyperactivity/impulsivity subscale was higher in the ADHD-C group (*t*_(21)_ = 2.45; *p* < 0.05).

The study, which was approved by the Ethics Committee of the Medical Faculty of the University of Erlangen–Nuremberg, was conducted in accordance with the Declaration of Helsinki. Children gave their assent and parents provided written informed consent.

### Procedure and task

In the testing session, children sat on a comfortable chair in front of a computer monitor (viewing distance: 72 cm). During EEG preparation, the children could watch age-appropriate films. The ANT, which consisted of four blocks of 48 trials each, lasted about 15 min (including short breaks between the task blocks). During the test brain electrical activity was recorded. The children received standardized instructions before performing a practice block of 24 trials. After each task block, a summary of the task performance was shown on the screen.

Children were instructed to “feed” a hungry fish that would appear above or below a fixation cross. If the fish pointed to the right (resp. left) side, the children had to press the right (resp. left) mouse button in order to feed the fish. This target fish was the center fish in a row of five fish with the flanking fish either looking in the same direction (congruent condition) or in the opposite direction (incongruent condition).

One of three cue conditions (equal probability) preceded the presentation of the fish: in the NeutralCue condition, an asterisk at the center of the screen indicated that the target fish was about to appear soon. In the SpatialCue condition, an asterisk was presented at the location of the target fish, indicating not only that the target was about to appear soon but also its location on the screen. In the NoCue condition, the fish were presented without a cue stimulus.

**Figure 1:**
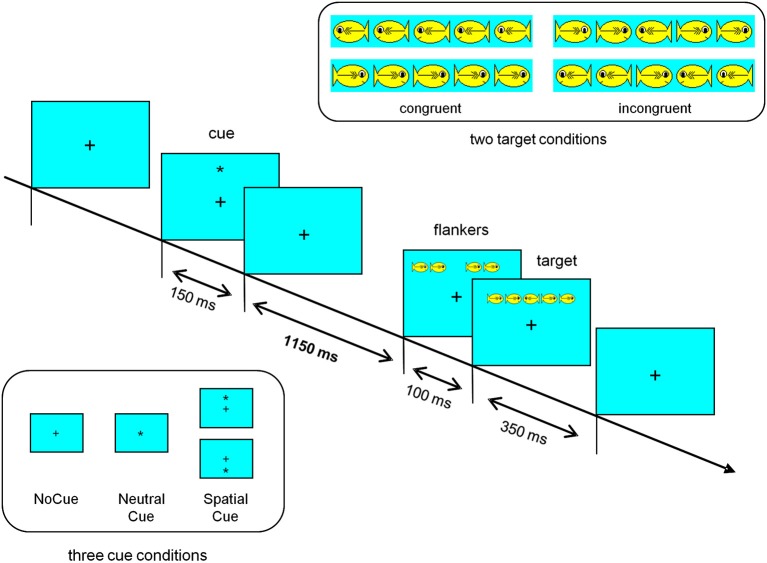
**Schematic illustration of the attention network test (ANT) as applied in our study**. A fixation cross located on the center of the computer screen was shown during the complete test. The row of hungry fish appeared above or below the fixation cross (about 1°). The target fish, i.e., the center fish, was visible for 350 ms. Presentation of the flanking fish started 100 ms before the center fish appeared. Cue stimuli were visible for 150 ms, starting 1400 ms before the target stimulus (fish). A cue stimuli was always followed by a target stimulus. In comparison to the original child version of the ANT (Rueda et al., 2004), a longer interval between cue and target stimulus was used (1400 ms instead of 600 ms) to elicit a contingent negative variation. Each fish subtended 1.6° of visual angle and the contours of adjacent fish were separated by 0.21°. The intertrial interval varied randomly between 3.5 and 5.0 s.

A schematic illustration of the ANT as applied in the present study, including technical details is presented in Figure 1. The test was realized in Presentation (Neurobehavioral Systems, Albany, CA, USA).

### EEG recording and preprocessing

A Brainamp recording system (Brainamp standard amplifier, Brain Products, Munich, Germany) was used. Brain electrical activity was recorded from 23 sintered Ag/AgCl electrodes (10/20 system; Fpz, Oz, mastoids). Positions for reference and ground electrode were FCz and CPz, respectively. Vertical and horizontal electrooculogram was recorded from electrodes placed above and below the right eye and at the outer canthi. A sampling rate of 500 Hz was used. Filter bandwidth at recording was 0.016–120 Hz. Impedances were kept below 20 kΩ.

For preprocessing and data analysis, the VisionAnalyzer software (Brain Products, Gilching, Germany) was used. After applying a 50 Hz notch filter and bandpass filtering (0.05–30 Hz, 24 dB/oct Butterworth filters), eye movement artifacts were corrected using independent component analysis (ICA, Jung et al., 2000). Signals were re-referenced to linked-mastoids. If amplitudes exceeded ±100 μV at any electrode, a segment of −300 to +700 ms around this artifact was excluded from further analyses.

EEG spectral analysis was conducted for NoCue segments of 1.5 s length, i.e., for segments before the onset of flanker stimuli which were not preceded by a cue stimulus. These were the segments with the longest “pure” EEG period without processing of cue or target stimuli and correspond to an attentive state.

For each child, at least 20 artefact-free segments (followed by a correct response to the target stimulus) had to be available. The number of segments without artefacts were slightly but not significantly smaller in the ADHD groups (control: 47.2 ± 9.6; ADHD-C: 44.3 ± 9.8; ADHD-I: 38.1 ± 14.6; *F*_(2,40)_ = 2.13, *p* = 0.13).

### Data analysis

The number of hits, median of reaction times and reaction time variability were determined. ANT-specific performance measures (alerting score, orienting score and conflict score; Fan et al., 2002) had not been significantly different for ADHD groups and control group in Kratz et al. (2011). So, for simplicity, they will be omitted in this manuscript. Reaction time measures were based on trials with correct responses. Only trials with reaction times between 200 and 1500 ms after target stimulus onset were included in the analysis.

**Figure 2:**
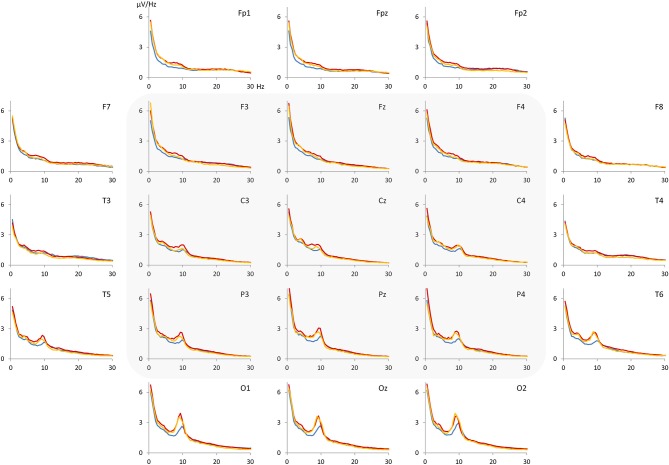
**Grand average spectra (voltage density) for control group (blue), ADHD-C group (red) and ADHD-I group (orange)**. The most pronounced differences between the groups appear to be in the range of 5.5–10.5 Hz. Electrodes F3, Fz, F4, C3, Cz, C4, P3, Pz and P4 were considered for statistical analysis.

For each NoCue trial, a voltage density spectrum was computed after applying a Hanning window and these spectra were averaged then. From the averaged spectra, voltage values for theta (4–7.5 Hz), alpha (7.5–12.5 Hz), beta (12.5–20 Hz) band as well as the theta/beta ratio were calculated at different electrodes (F3, Fz, F4, C3, Cz, C4, P3, Pz, P4). In Figure 2, the grand average spectra for control, ADHD-C and ADHD-I groups are depicted. Based on visual inspection, the largest differences between the groups seem to occur within an upper-theta/lower-alpha (5.5–10.5 Hz) band. Therefore, we decided to consider this band in addition to the traditional EEG bands.

### Statistical analysis

Performance data (hits, reaction time measures) were analyzed using a one-way ANOVA with between-subject factor GROUP (control, ADHD-C, ADHD-I). *t*-tests were applied for *post hoc* analysis (pairwise comparisons of two groups) using Bonferroni-Holm correction to control for multiple comparisons.

For the different EEG frequency bands, repeated-measure ANOVAs were computed with between-subject factor GROUP and repeated-measurement (electrode) factors Y (frontal [F3, Fz, F4], central [C3, Cz, C4], parietal [P3, Pz, P4]) and X (left [F3, C3, P3], midline [Fz, Cz, Pz], right [F4, C4, P4]) to test potential topography/laterality effects. *Post hoc* analysis also comprised correction for multiple comparisons (Bonferroni-Holm). Corrected *p*-values are reported.

Associations between EEG spectral parameters and performance measures were studied focusing on those EEG measures for which largest group-specific effects were obtained in the before-mentioned analysis. We controlled for age-related effects. However, as the portion of 8 year-old children was higher in the ADHD groups, controlling/correcting for age-related changes by considering the complete sample would lead to an overestimation of age-related changes at the cost of group-related effects. Instead, we decided to correct for age-related changes in the complete sample based on the regression coefficient of the control group. Pearson’s correlation coefficients were calculated for potentially age-corrected measures. If significant correlations were found for the complete sample, we also tested ADHD groups separately to exclude spurious correlations.

IBM SPSS Statistics (Version 20.0) was used for statistical analysis.

## Results

### Performance measures

Results of performance measures are summarized in Table 1. Reaction time variability was significantly higher in the two ADHD groups compared to the control group (control vs. ADHD-I: *t*_(26)_ = −3.32; *p* (corr.) = 0.009; control vs. ADHD-C: *t*_(32)_ = −2.61; *p* (corr.) = 0.03). For the median of reaction times, a GROUP effect was obtained due to higher reaction times in the ADHD-I group in comparison to the control group (control vs. ADHS-I: *t*_(26)_ = −3.71; *p* (corr.) = 0.003) as well as the ADHD-C group (ADHD-C vs. ADHD-I: *t*_(22)_ = −2.36; *p* (corr.) = 0.05).

### Spectral EEG parameters

**Figure 3:**
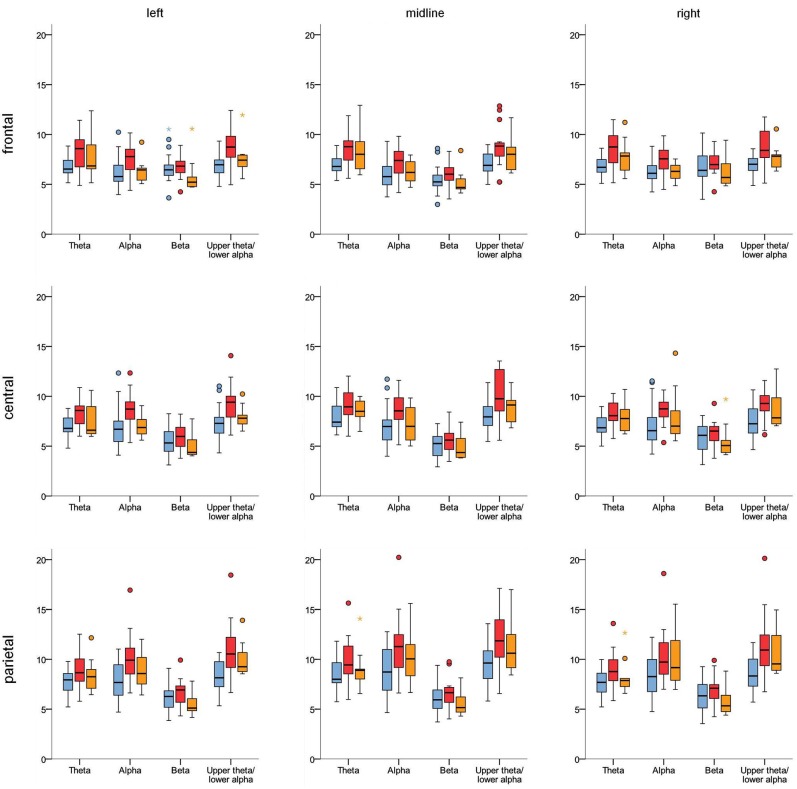
**Boxplots illustrating the topography of EEG activity in the different frequency bands for control group (blue), ADHD-C group (red) and ADHD-I group (orange)**. Unit: μV.

Results of the ANOVAs for the different frequency bands are summarized in Table 2. For all frequency bands considered, the repeated-measure ANOVAs revealed (highly) significant effects for the within-subject factors X, Y and their interaction X^*^Y related to the topography of the EEG activity in the different frequency bands. Theta, alpha and 5.5–10.5 Hz activity were highest at electrode Pz (parietal, midline); see also Figure 3. The highest beta activity was measured at left and right frontal electrodes (F3 and F4). The theta/beta ratio had its maximum at electrode Cz. No significant interaction effect containing the factor Group was obtained, i.e., topography did not differ significantly between the groups.

**Table 2:** **EEG measures and statistical results**.

	**Children with ADHD**		**Controls (*N* = 19)**	**Statistics (repeated-measure ANOVAs)**
	**ADHS-C (*N* = 15)**	**ADHS-I (*N* = 9)**		
Theta (3.5–7.5 Hz)	8.73 ± 1.72	8.22 ± 1.65	7.37 ± 1.14	G: *F*_(2,40)_ = 3.7, *p* = 0.034; part. *η*^2^ = 0.16 X: *F*_(2,80)_ = 67.3, *p* < 0.001; part. *η*^2^ = 0.63 Y: *F*_(2,80)_ = 20.4, *p* < 0.001; part. *η*^2^ = 0.34 X*Y: *F*_(4,160)_ = 4.9, *p* = 0.002; part. *η*^2^ = 0.11
Alpha (7.5–12.5 Hz)	8.92 ± 1.93	7.85 ± 1.40	7.16 ± 1.83	G: *F*_(2,40)_ = 4.0, *p* = 0.026; part. *η*^2^ = 0.17 X: *F*_(2,80)_ = 6.1, *p* = 0.004; part. *η*^2^ = 0.13 Y: *F*_(2,80)_ = 80.4, *p* < 0.001; part. *η*^2^ = 0.67 X*Y: *F*_(4,160)_ = 15.2, *p* < 0.001; part. *η*^2^ = 0.28
Upper-theta/lower alpha (5.5–10.5 Hz)	9.96 ± 2.34	9.01 ± 1.32	7.78 ± 1.42	G: *F*_(2,40)_ = 6.3, *p* = 0.004; part. *η*^2^ = 0.24 X: *F*_(2,80)_ = 26.1, *p* < 0.001; part. *η*^2^ = 0.40 Y: *F*_(2,80)_ = 61.6, *p* < 0.001; part. *η*^2^ = 0.61 X*Y: *F*_(4,160)_ = 11.1, *p* < 0.001; part. *η*^2^ = 0.22
Beta (12.5–20 Hz)	6.46 ± 1.18	5.55 ± 1.37	5.98 ± 1.40	G: *F*_(2,40)_ = 1.4, n.s.; part. *η*^2^ = 0.07 X: *F*_(2,80)_ = 74.1, *p* < 0.001; part. *η*^2^ = 0.65 Y: *F*_(2,80)_ = 21.6, *p* < 0.001; part. *η*^2^ = 0.35 X*Y: *F*_(4,160)_ = 16.5, *p* < 0.001; part. *η*^2^ = 0.29
Theta/beta ratio	1.40 ± 0.29	1.55 ± 0.39	1.30 ± 0.27	G: *F*_(2,40)_ = 2.1, n.s.; part. *η*^2^ = 0.09 X: *F*_(2,80)_ = 184.8, *p* < 0.001; part. *η*^2^ = 0.82 Y: *F*_(2,80)_ = 23.3, *p* < 0.001; part. *η*^2^ = 0.37 X*Y: *F*_(4,160)_ = 11.0, *p* < 0.001; part. *η*^2^ = 0.22

*For control and ADHD groups, the group’s mean (for the average regarding the different EEG frequency bands over frontal, central and parietal electrodes considered in the ANOVAs) ± SD is presented. Unit (except theta/beta ratio): µV. For the repeated-measure ANOVAs, the results obtained for the between-subject factor Group (G), the within-subjects factors X (left, midline, right) and Y (frontal, central, parietal) and their interaction are provided*.

For theta activity, alpha activity and particularly upper-theta/lower-alpha (5.5–10.5 Hz) activity, significant group main effects indicated higher activity in the ADHD groups. *Post hoc* analysis revealed that 5.5–10.5 Hz activity was higher particularly in the ADHD-C group (control vs. ADHD-C: *t*_(32)_ = −3.35; *p* (corr.) = 0.006) and to a smaller extent in the ADHD-I extent in the ADHD-I group (control vs. ADHD-I: *t*_(26)_ = −2.17; *p* (corr.) = 0.08, *p* (uncorr.) = 0.04).

No significant main effect for the theta/beta ratio was found. However, a medium effect size (part. *η*^2^ = 0.09) may indicate some effect which did not turn out to be significant due to the limited sample size. So, we decided to look at the theta/beta ratio in more detail in an exploratory way. At least a tendency for a higher theta/beta ratio (averaged over the nine electrodes) in the ADHD-I group (control vs. ADHD-I: *t*_(26)_ = −2.0; *p* = 0.057; Cohen’s *d* = 0.8) was obtained whereas no effects were observed for the ADHD-C group (control vs. ADHD-C: *t*_(32)_ = −1.02; n.s.). When group at single electrodes, significant effects were found for electrodes F3 and Fz (control vs. ADHD-I: *t*_(26)_ ≤ −2.28; *p* ≤ 0.03).

### Associations between spectral EEG parameters and performance measures

**Figure 4:**
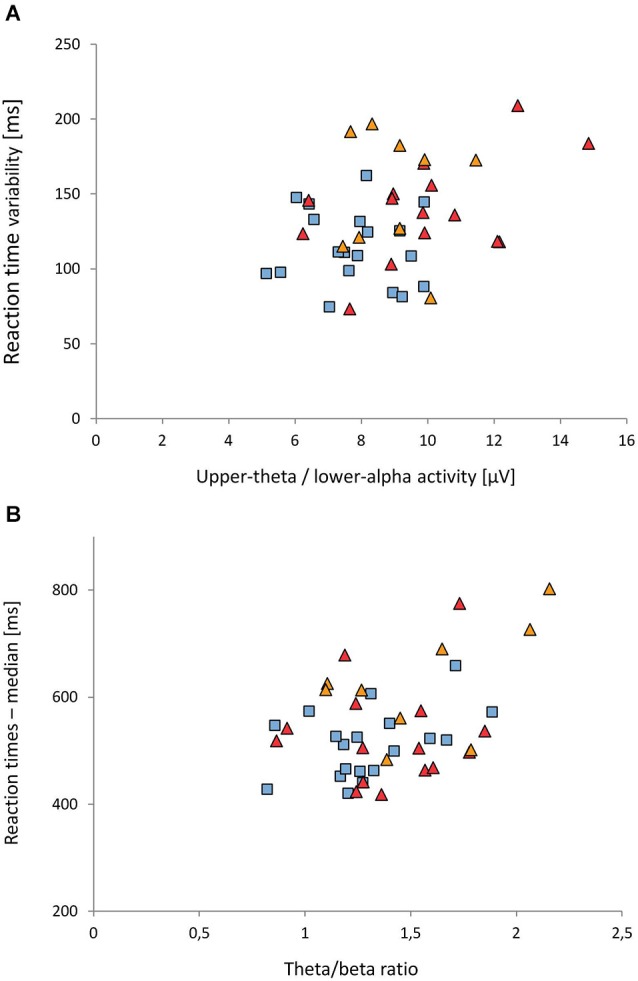
**Associations between EEG and reaction time measures. (A)** Upper-theta/lower-alpha (5.5–10.5 Hz) activity vs. reaction time variability. **(B)** Theta/beta ratio vs. median of reaction times. Reaction time measures were adjusted for developmental effects (for details see text). Control group: blue rectangles; ADHD-C group: red triangles; ADHD-I: orange triangles.

As only GROUP main effects were found in the ANOVAs, we considered the average of all electrodes for the correlational analysis. A significant correlation was found between the activity in the 5.5–10.5 Hz band (averaged over frontal, central and parietal electrodes) and reaction time variability (*r* = 0.34, *p* = 0.025): Higher activity in the 5.5–10.5 Hz band was associated with higher reaction time variability (see Figure 4A). For the ADHD-C group, the correlation coefficient was 0.48.

A significant correlation was also obtained between the theta/beta ratio (averaged over frontal, central and parietal leads) and the median of reaction times (*r* = 0.42, *p* = 0.005); see Figure 4B. The higher the theta/beta ratio was, the longer reaction times were. This effect was most prominent in the ADHD-I group (*r* = 0.54).

Hence, significant associations were found for those frequency bands and performance (reaction time) measures with deviations in the ADHD groups. It has to be noted that these correlations did not reach statistical significance in the respective ADHD group due to the small group sizes.

## Discussion

In the present study, we conducted EEG spectral analysis during an attention demanding period in children with ADHD (compared to typically developing controls). Deviant EEG patterns were obtained with subtype-specific differences between the DSM-IV combined type and the predominantly inattentive subtype.

### Spectral EEG measures during an attentive state in ADHD (subtypes)

In contrast to recent resting-EEG studies (e.g., Ogrim et al., 2012; Liechti et al., 2013), significant differences related to the theta band and the alpha band were obtained between children with ADHD and typically developing children: activity in these frequency band was significantly larger in children with ADHD. In the ADHD-C group, effects were most prominent when considering the 5.5–10.5 Hz (upper-theta/lower-alpha) band. Global statistical analysis did not reveal a significant group effect for the theta/beta ratio, i.e., the major part of the children with ADHD was not characterized by an increased theta/beta ratio. On the other hand, a large effect size for the comparison of control and ADHD-I group may indicate an increased theta/beta ratio in children of the predominantly inattentive subtype comparable to the findings of Buyck and Wiersema (2014) obtained in the resting EEG. However, this finding is limited by the rather small size of our ADHD-I group.

As inattention scores of the German ADHD rating scale were comparable for ADHD-C group and ADHD-I group we argue that the differential pattern does not reflect different severity of inattention symptoms but rather suggest that there are different neural mechanisms accounting for attentional dysfunctions in ADHD subtypes. In Heinrich et al. (submitted), we had already reported different distributions of cue-P3 single trial amplitudes for the two ADHD groups further strengthening this point of view.

### Associations between spectral EEG parameters and reaction time measures

Interestingly, significant (positive) correlations between those spectral EEG parameters and reaction time measures (5.5–10.5 Hz activity and reaction time variability; theta/beta ratio and median of reaction times) were found for which differences between the ADHD groups and the control group had been obtained. These associations suggest a functional relevance of the EEG parameters, particularly in the context of ADHD: a suboptimal neural state at stimulus presentation results in impaired performance. As we controlled for age effects and also considered the ADHD groups separately, it seems rather unlikely that the correlations obtained for our data reflect spurious correlations.

Loo and Smalley (2008) had also reported a positive correlation between reaction time variability and activity in the theta and alpha band during an attention (continuous performance) test. Increased reaction time variability is a robust finding in children with ADHD with medium to large effect sizes being reported (meta-analysis for example in Kofler et al., 2013: Hedges’ *g* = 0.76). Increased activity in the upper-theta/lower-alpha band, which may be interpreted as an underactivated neural state, could reflect a neural mechanism underlying increased reaction time variability in ADHD besides top-down control and motor preparation processes (Karalunas et al., 2014). It seems unlikely that slower reaction times in the ADHD-I group of our sample are mainly due to very slow reaction times in a few trials but they rather reflect a generally slower processing/response style. Findings indicate that this slowing may be related to a higher theta/beta ratio. The differential associations regarding ADHD-C and ADHD-I groups further support the notion of distinct neural mechanisms underlying attentional dysfunctions in ADHD subtypes.

### Potential implications for neurofeedback training in ADHD

NF may be interpreted as an approach to gain self-control over a certain aspect of neural activity associated with a specific cognitive or emotional state (Gevensleben et al., submitted). In this respect, findings of the present study may have the following implications for NF training in ADHD.

In children with ADHD of the combined type, an upper-theta/lower-alpha (5.5–10.5 Hz) protocol associated with an attentive state may be more effective than theta/beta training. It will have to be studied if indication criteria for the use of a specific protocol based on (inter alia) EEG characteristics at pre-training can be developed. As theta activity in the resting EEG at pre-training was found to be a predictor for the effects of theta/beta training (Gevensleben et al., 2009b), this seems to be a realistic task.

Up to now, only a single EEG channel is typically used to calculate feedback information in EEG NF training. For theta/beta training in ADHD, most often electrode Cz is considered. In our data, increased upper-theta/lower-alpha activity in the ADHD-C group and a higher theta/beta ratio in the ADHD-I group were not topographically specific, i.e., they were not restricted to/particularly pronounced at a certain electrode. Looking at single electrodes, effects at electrode Cz appeared rather smaller than larger compared to frontal, electrodes (F3, Fz).

It has to be taken into consideration that frontal midline theta (associated with working memory and cognitive control processes; Jensen and Tesche, 2002; Enriquez-Geppert et al., 2014) could interfere with the more generalized theta pattern addressed for example in theta/beta training if feedback information is calculated from Cz only. So, in our opinion, a more robust/more specific feedback signal may be obtained if not a single channel but a combination of several electrodes is used. If NF training does not target a topographically specific EEG pattern, the average of a grid of distributed electrodes may be preferable.

NF training trials may also be combined with attention tasks to facilitate training effects at the performance level: depending on the protocol applied, faster or less variable reaction times may be achieved. Regarding other tasks (e.g., reading, listening), it will have to be tested whether refined frequency bands and feedback parameters, respectively, may also be more characteristic for children with ADHD.

### Limitations of our study

Findings are limited by the generally small sample size. However, we’d see sample size more critical if findings had not turned out to be significant. Large effect sizes were obtained and effects were not just due to outliers suggesting clear differences in the distribution of control and ADHD groups. In any case, larger samples will have to be studied to see if results are confirmed and to what extend EEG-based subtypes can be found.

We could not compare resting and active EEG conditions directly. Thus, it cannot be excluded that corresponding effect sizes could have also been found in the resting EEG of our sample. However, in our opinion, our findings complement/are compatible with results of recent studies that either report no significant global differences in recent resting EEG studies (e.g., Liechti et al., 2013; Buyck and Wiersema, 2014) and/or more pronounced effects in active compared to resting conditions (e.g., Loo and Smalley, 2008).

## Conclusions

During an attentive state, children with ADHD are characterized by an underactivated state in the EEG with subtype-specific differences. Whereas the most prominent effect was obtained for the upper-theta/lower alpha (5.5–10.5 Hz) range in children of the combined type, hints for an increased theta/beta ratio were found in children of the predominantly inattentive subtype. The functional relevance of these EEG parameters was indicated by associations with reaction time measures, which were pronounced most in the ADHD groups. Findings may provide a rationale for applying NF training protocols targeting theta activity and theta/beta ratio in subgroups of children with ADHD to achieve an attentive state. In this respect, it will be interesting if indication criteria for a specific protocol in an individual child can be developed which can be applied in clinical practice.

## Conflict of interest statement

The authors declare that the research was conducted in the absence of any commercial or financial relationships that could be construed as a potential conflict of interest.

## Footnotes

^1^In the fourth edition of the Diagnostic and Statistical Manual of Mental Disorders (DSM-IV; American Psychiatric Association, 2000), three subtypes of ADHD (combined type, predominantly inattentive type, predominantly hyperactive-impulsive type) are distinguished reflecting the heterogeneity of the disorder. In clinical practice, the combined type appears to be most common. Besides the combined type, research focuses also on the predominantly inattentive type.

^2^A reduced CNV is typically considered as a rationale to apply training of slow cortical potentials (SCPs) in children with ADHD (Heinrich et al., 2007).
